# Supplementary figures and images for: ‘Candidatus Phytoplasma ziziphi’ Changes the Metabolite Composition of Jujube Tree Leaves and Affects the Feeding Behavior of Its Insect Vector Hishimonus hamatus Kuoh
Source: Insects. 2023 Sep 6;14(9):750. doi: 10.3390/insects14090750 (PMC10531678; doi:10.3390/insects14090750)

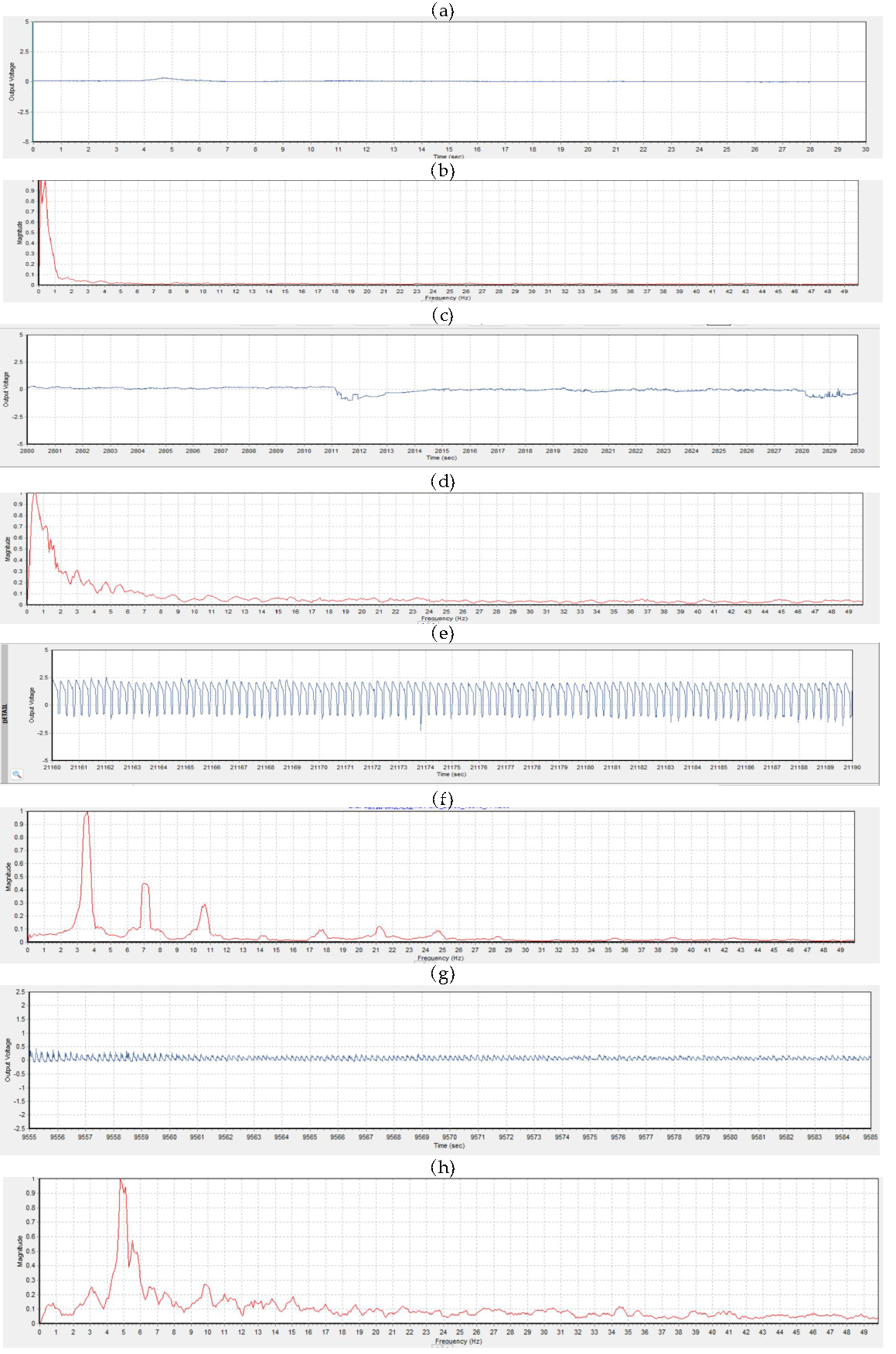

Supplement: Supplementary file 1 [file insects-14-00750-s001.zip › Figure S1 波形图整合.png]
